# Acute drug induced hepatitis secondary to a weight loss product purchased over the internet

**DOI:** 10.1186/1475-2891-6-13

**Published:** 2007-06-27

**Authors:** Deepak Joshi, Tim JS Cross, Voi Shim Wong

**Affiliations:** 1 The Whittington Hospital, Highgate Hill, London, N19 5NF, UK

## Abstract

**Background:**

Many people now seek alternative methods of weight loss. The internet provides a readily available source of weight reduction products, the ingredients of which are often unclear. The authors describe a case of acute hepatitis in a 20 year old woman caused by such a product purchased over the internet.

**Case Presentation:**

A 20-year old woman presented with a two day history of abdominal pain, vomiting and jaundice. There were no identifiable risk factors for chronic liver disease. Liver function tests demonstrated an acute hepatitis (aminoaspartate transaminase 1230 IU/L). A chronic liver disease screen was negative. The patient had started a weight loss product (*Pro-Lean*), purchased over the internet two weeks prior to presentation. The patient was treated conservatively, and improved. The sequence of events suggests an acute hepatitis caused by an herbal weight loss product.

**Conclusion:**

This case report highlights the dangers of weight loss products available to the public over the internet, and the importance of asking specifically about alternative medicines in patients who present with an acute hepatitis.

## Background

Obesity is increasingly recognised as a medical problem [1]. Many people now seek alternative methods of weight loss, in particular weight reduction products, some of which are available over the internet. The potential dangers of these products are often not known. The authors describe a case of acute hepatitis in a 20 year old woman caused by such a product purchased over the internet.

## Case Presentation

A 20-year-old woman presented with a two-day history of right upper quadrant pain, vomiting and jaundice. She did not drink alcohol and her only regular medication was the oral contraceptive pill that she had taken for many years. There was no history of recent foreign travel. There had also been no recent use of paracetamol, non steroidal anti-inflammatory drugs (NSAIDs), anti-biotics, or recreational drugs. She denied the use of intravenous drugs. On examination, she was icteric, with epigastric tenderness but no palpable organomegaly. There was no hepatic encephalopathy, nor stigmata of chronic liver disease. The full blood count was normal; white cell count 7.4 10^9^/L (NR 4–11), platelets 200 10^9^/L (NR 150–400), haemoglobin 15.9 g/dl (NR 11–13). Urea and electrolytes and coagulation studies were also normal. Liver functions tests (LFTs) were abnormal; bilirubin 53 μmol/L (1–19), alkaline phosphatase 92 IU/L (ALP 25–100), aspartate aminotransaminase 1230 IU/L (AST 7–40), albumin 40 g/L (36–52), and international normalised ratio 1.1 (INR 0.8–1.3). Hepatitis A IgM (HAV IgM), hepatitis B surface antigen (HBsAg) and hepatitis C antibody (HCV Ab) were negative. Herpes simplex virus, Epstein Barr virus and cytomegalovirus were all undetectable. Auto-immune antibody serology, serum copper, caeruloplasmin, and ferritin were normal. Hepato-biliary ultrasonography was normal. On further questioning, the patient volunteered that she had started a weight-loss product called, Pro-lean, two weeks before admission. This had been purchased over the internet. Pro-lean was stopped, and the LFTs started to improve. On discharge (5 days later) her LFTs continued to improve (see Table 1). A liver biopsy was not performed, because of the rapid biochemical improvement. At two-months from the initial presentation the LFTs had normalised (Table 1), and the patient remained well. Repeat virology (HAV IgM, HBsAg and HCV Ab), in the convalescent stage remained negative.

**Table 1:** Progression of liver function tests.

	**Admission**	**Discharge**	**2 months post discharge**
**Bilirubin μmol/L (1–19)**	53	42	8
**ALP IU/L (25–100)**	92	101	35
**AST IU/L (7–40)**	1230	286	28
**Albumin g/L (36–52)**	40	38	38
**INR (0.8–1.3)**	1.1	1.0	1.1

## Discussion

Two-thirds of adults over 45 years are now classified as being obese. Obesity was estimated to have cost the NHS £500,000,000 in 1998. As levels have risen, so to have the number of diets and treatments alternative to the traditional methods of weight loss.

Pro-Lean, by Pro-image is a weight loss product containing herbs, botanicals and chromium. One capsule (to be taken once per day) contains 150 μg of chromium (chromium dinicotinate glycinate), 12–15 μg of vitamin B12, 50 μg of vanadium, caffeine 200 mg, 150 μg of cyperus root extract and 50 μg of L-tyrosine. Furthermore, other ingredients include ma-huang, guarana, kola nut, white willow bark, ginkgo biloba, bladderwrack, gotu kola, boron, ginseng, fo-ti, magnesium salicylate, folic acid, bee pollen, spirulina and ginger root. Chromium toxicity has been linked with cases of hepatic, renal and cardiac failure, as well as bronchial malignancy [2]. The daily requirement is 5 to 115 μg/day. Chromium-induced toxic hepatitis has previously been described, in a 35-year old woman who had been taking a chromium dietary product (200 μg/day) for five months [3]. However, it is often difficult to determine which component of these products is responsible for hepato-toxicity as there are often many pharmacologically active constituents. Many herbal products have been implicated in liver toxicity including kava, chapparal, germander, comfrey roots [4], and also ma huang [5]. Orthotopic liver transplantation has been required in some cases of drug induced hepatitis caused by herbal remedies observed [6]. In the United Kingdom health products are treated as food if they are not granted a medical license. No health claims can be made on the labelling. In the United States food, and drug administration (FDA) approval is only needed for medicines. Herbal products do not fall into this category and are viewed as dietary supplements. Patients may regard these products as healthy alternatives and may be unaware of their side effects, and contents. An analysis of 260 Asian patent medicines studied by the Californian health services, food and drug branch found that 32% of the analysed samples contained undeclared pharmaceuticals and heavy metals [7].

On discontinuation of Pro-Lean the patient improved both clinically and bio-chemically, suggesting causality. The clinical diagnostic scale (CDS) is a useful screening tool which has been developed for the diagnosis of drug induced liver injury [8]. The CDS score in this case was 12. A CDS score of >9 is assumed to be drug related unless an alternative diagnosis is suspected. It is difficult to ascribe the observed hepatic toxicity to one specific ingredient alone contained within Pro-Lean. Chromium toxicity would be less likely given the normal renal function. The clinical presentation and pattern of hepatic toxicity in this case is very similar to the case described by Nadir et al (1996), suggesting ma huang may have played an active role.

## Conclusion

This case highlights the importance of inquiring into alternative medicines, herbal remedies and unconventional diets in cases of acute hepatitis, where the cause is uncertain. It also raises questions regarding the regulation, licensing and safety of herbal and alternative health products that can be bought over the internet.

## Competing interests

The author(s) declare that they have no competing interests.
